# Urban health issues of the marginalised in Dar es Salaam, Tanzania: A resonant voice calling for action

**DOI:** 10.7189/jogh.13.03060

**Published:** 2023-10-27

**Authors:** Kyoung Kyun Oh, Rashid S Mfaume, Uless A Mbise, Ntuli A Kapologwe

**Affiliations:** 1 Korea Foundation for International Healthcare (KOFIH) Tanzania Office, Dar es Salaam, Tanzania; 2 Regional Commissioner’s Office, Dar es Salaam, Tanzania; 3 Health, Social Welfare and Nutrition Services, President’s Office – Regional Administration and Local Government, Dodoma, Tanzania

**Figure:**
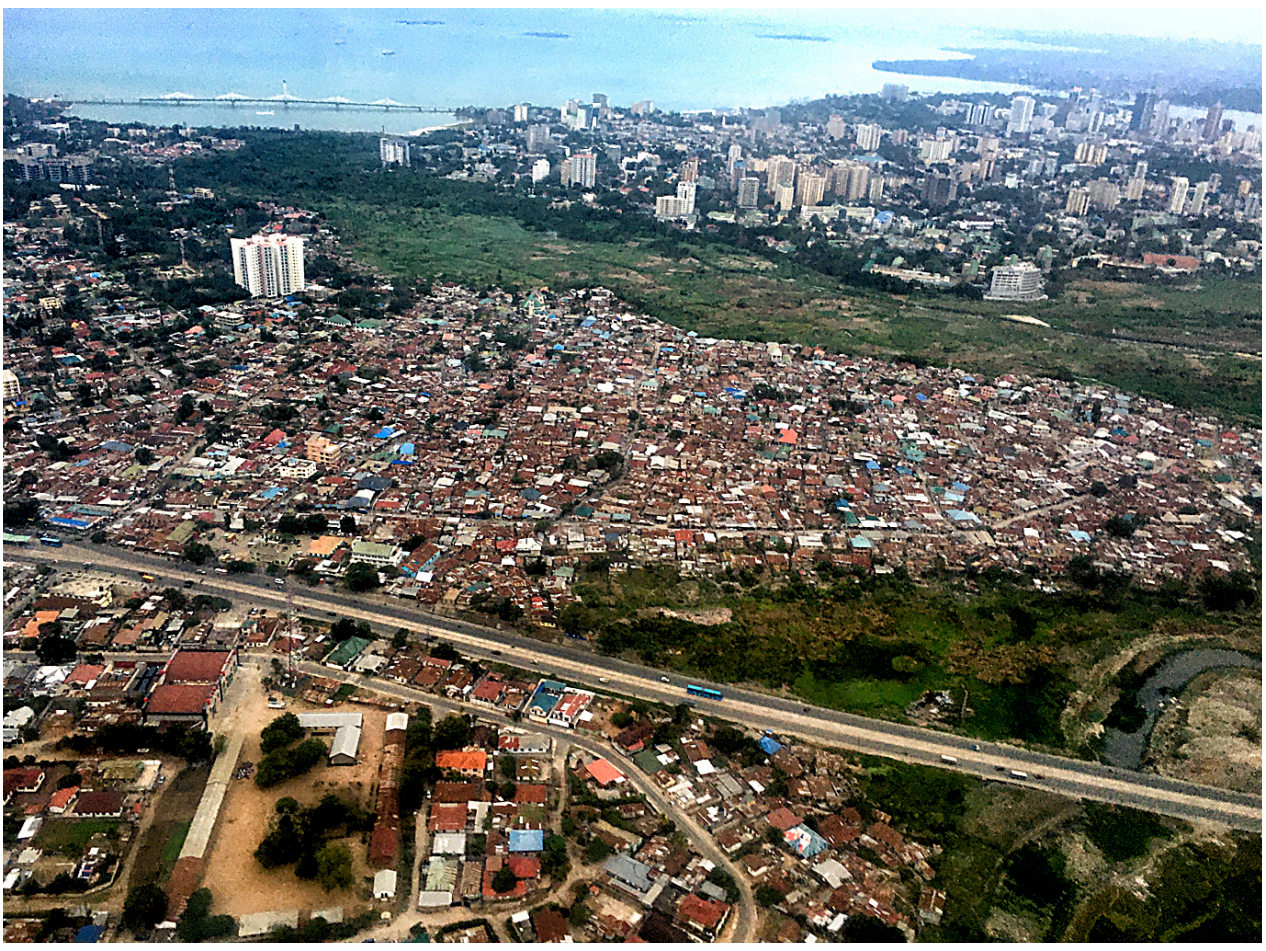
Photo: An aeroplane view of the unequal types of settlement in Dar es Salaam Source: The photo was taken by the corresponding author on 15 July 2022.

## URBANISATION AND PUBLIC HEALTH ISSUES

Urban areas cannot be defined in a word, but they are undoubtedly unique spaces because of their bipolar characteristics. On one side, urban areas have a high potentiality with a talented workforce, a faster-growing population, and a greater concentration of wealth. The other side has problems with more significant inequity, inequalities, higher levels of unemployment, and a disproportionate geopolitical and economic power [1,2]. Due to such characteristics, in developing settings, including Tanzania, internal migrations from rural to urban areas to seek better job opportunities, well-being and prosperity in the growing cities are common phenomena. They are even expected to increase over the next decades due to economic development. As a result, all goods and services are inevitably more concentrated in urban areas. This leads to these spaces generating other goods and socio-economic services in a secondary or tertiary way. Hence, the urban can be defined as a core political and socio-economic power collective.

## HEALTH AND DEMOGRAPHIC INFORMATION OF DAR ES SALAAM

Dar es Salaam is the fifth-largest city in Africa and is expected to become a mega-city (with more than ten million residents) by 2030 [3,4]. Among 26 regions in Tanzania’s Mainland, Dar es Salaam, the biggest city, is home to more than five million people [5]. This number accounts for more than 10% of the total population of Tanzania. The city’s population growth rate is 7% yearly, more than twice the national growth rate of 3% on average [3,4]. The majority of the city’s residents are younger than in other regions. Further, 66% of the city population is aged between 15 and 64 years [5]. The average of women of reproductive age is 62%, approximately 11% points higher than the national average of 51%. There are 572 health facilities registered in Dar es Salaam; amongst them, 42 are hospitals, 48 health centres, and 423 dispensaries [5]. Health services depend mainly on private rather than public health services. For example, only 19% (111 out of 572) of health facilities account for public health service provision [5]. Given that the national coverage of public health services is approximately 70%, this number is absurdly low [5]. Even if health facilities are easily accessible, the health system is heavily private-oriented. Thus, less resilient social classes cannot access quality care due to financial insufficiency. This mismatch leads to the fact that urban dwellers can easily access health services, but not all health outcomes are better for urban inhabitants than for rural inhabitants. So, the rapidly increasing population has allegedly pressured social services, including maternal-child and adolescent health services [4,6]. For example, although Dar es Salaam is considered to have more educated and better-off communities and a closer network of health facilities, it does not appear to be the leading one in terms of essential health care services [3-5]. Urban life seems to be healthy for those who are in middle and high-income quantile/groups only.

## INFORMAL SETTLEMENTS IN DAR ES SALAAM

Like other fast-growing cities, Dar es Salaam is challenged by the increasing number of informal settlements due to rapid unplanned urbanisation. The movement of people from all over the country has resulted in pressure on the social services provision, which is not in keeping with the increase in the number of people. Moreover, the exact number of migrants and dwellers in the city is not well-tracked or updated by the local authorities [3]. There is scarce reliable data on where these migrants usually settle down in the early stages of living in urban areas and where they seek their social services. There are areas which are considered to have the majority of informal settlement are Kigogo, Buguruni, Msimbazi Valley, Tandale, Manzese, Mbagala, Vingunguti, and Temeke, which are very densely populated with no sound sewage systems and share some characteristics of some places like that of Kibera in Kenya which is believed to be the largest urban slum in Africa [5,7].

## HEALTH BOTTLENECKS IN INFORMAL SETTLEMENTS IN DAR ES SALAAM

Not only the midterm review of the Health Sector Strategic Plan IV but also the Health Sector Strategic Plan V have highlighted the poor health outcomes in urban areas and the urban poor in Dar es Salaam [5]. The Health Sector Strategic Plan V has proposed that “the health sector recognise Dar es Salaam as a specific health zone” to fulfil the health demand and need in urban health care [5]. It also points out that child and adult health services at the primary health care level in urban poor are needed to create equity. Health indicators such as life expectancy, stunting, diarrheal disease, human immunodeficiency virus (HIV)/acquired immunodeficiency syndrome (AIDS), and under-five mortality are worse than its cohorts in urban and rural areas in Tanzania [8,9] (**Table 1**).

**Table 1:** A comparison table for health indicators among urban, rural and urban informal settlement dwellers [5]

Health indicator*	Urban	Rural	Urban informal settlement
Life expectancy (years)	60	62	44-46
HIV/AIDS (%)	7.2	4.3	No data
Unimproved latrine (%)	21	73	83
No latrine (%)	2	13	14
Under five mortality/1000 live births	71.2	65.9	97
Low birth weight (%)	9.1	5.8	No data
Stunting (%)	24.7	37.8	44-56
Diarrhoea (%)	14.1	11	60
Prolateral feeds (%)	12	14.5	91
Maternal mortality ratio	432	336	No data
Overweight (%)	42	21	No data

HIV – human immunodeficiency virus, AIDS – acquired immunodeficiency syndrome

*The authors have modified the table from the Health Sector Strategic Plan V July 2021-June 2026. Generally, health indicators in urban areas are better than in rural areas. However, there seems to be further gaps between urban areas and informal settlements rather than between urban and rural areas.

Three different bottlenecks are suggested at the different levels. The first bottleneck is at the facility level itself. This is not only concerned about the scarce number of health facilities but also the imperfect quality of health. Lack of health service delivery, including absences of personnel, inadequate skill levels, lack of drugs and basic medical equipment, and capacity of service provision at health facilities in informal settlements/slum areas, results in poor health outcomes. The second bottleneck is at the community level. Community people are socio-politically powerful when they mainstream their health issues, take an active role, and increase their voices by participating in decision-making [3]. However, people living in informal settlements are often negligible since their political voices and socio-economic power are too trivial to echo political leaders [10,11]. People often lack information and support in the coalition of political voices. The last bottleneck is governance. Most unplanned areas generally have no address. Since the address is a basic unit for regularisation, no address means that the area is masked from the political and social governing system [12]. Internally displaced persons are not reflected in statistics, leading to blind spots in which authorities cannot take appropriate actions for these individuals. Above all, the government should have been responsible for the social determinants of health, such as poverty, income, neighbourhood and social dynamics in dealing with urban slums. It emphasises the role of facilitating the appropriate interventions in the health field bottlenecks. Simultaneously, it is reaffirmed that authorities are responsible for future studies and surveys, which should provide disaggregated data in the health sector regions in Dar es Salaam.

## CONCLUSION

Urbanisation, especially for those living in informal settlements, requires new health care delivery models and improved accountability for the roles of public health services and the relationship between the government and private sector providers. Like other resource-limited settings, Tanzania cities inherently offer inequitable health services due to many challenges, including social infrastructures. Solving health issues needs to be orchestrated in harmonisation with other sectors. Lens of health-in-all-policies to protect and improve the health of urban dwellers and maintain a healthy environment are important. Since urban health issues for the vulnerable are related to health geographics, politics, economy, planning, society, engineering and mechanics, a comprehensive strategy which deals with multi-cooperative, multi-sectoral, dimensional, and consilient approaches needs.

Tanzania has been implementing several health sector reforms to improve health outcomes and equity in the different segments of the population. One of the notable reforms is the introduction of the Primary Health Care Development Program (PHCDP) (2007-2017), which aimed at geographically targeted construction and equipping of the primary health facilities to create equity in health service delivery [13]. Another reform was the Direct Health Facility Financing (DHFF) in 2017 and 2018, which was part of implementing financial decentralisation that focused on increasing equity and autonomy in financial use at the primary health care level [14]. The introduction of DHFF has been coupled with adjustment of the resource allocation formula for disbursement of funds at the lower level, which contains adjustors, namely, poverty index, land capped factor, under five mortalities and service utilisation at the particular facility [14]. Despite these reforms, there has still been inequitable access to quality health services among residents of urban areas, resulting in the epidemiologic shift in the disease burden of certain communicable and non-communicable diseases [15,16].

Interventions carried out by external funders (e.g. Official Development Assistance), such as budget support, technical support, health projects and programmes, should specifically be functioning to fill the gap in health inequity and inequality. This is not enough to be just based on the recipient-centric perspective and cooperative partnership between donor and recipient; it needs to reflect on how to sophisticatedly deal with the health disputes in the context of the specific strategy and roadmap. In this aspect, mainstreaming urban health issues among the vulnerable would fulfil both conditions, increasing health equity and preparedness for future pandemics. Suppose there are not enough interventions, awareness, and attention to health issues in urban slums in the fast-growing area of the world. What if we are the bystanders in this issue, there might be other types of social, political, and biological pandemics that begin here.

## References

[1] Dijkstra L, Hamilton E, Lall S, Wahba S. How do we define cities, towns, and rural areas. 2020. In: Sustainable Cities. WORLD BANK BLOGS [Internet]. Washington, D. C.: The World Bank. C2023. Available: https://blogs.worldbank.org/sustainablecities/how-do-we-define-cities-towns-and-rural-areas. Accessed: 7 Jul 2022.

[2] United Nations Department of Economic and Social Affairs (UN DESA). 68% of the world population projected to live in urban areas by 2050, says UN. Available: https://www.un.org/sw/desa/68-world-population-projected-live-urban-areas-2050-says-un. Accessed: 7 July 2022.

[3] Msuya I, Moshi I, Levira F. Dar es Salaam: the unplanned urban sprawl threatening neighbourhood sustainability. Available: http://www.centreforsustainablecities.ac.uk/wp-content/uploads/2020/10/SHLC_Research_Summary_11_DAR_ES_SALAAM.pdf. Accessed: 22 July 2022.

[4] Todd G Msuya I Levira F Moshi I City profile: Dar es Salaam, Tanzania. Environ Urban Asia. 2019;10:193-215. 10.1177/0975425319859175

[5] Government of the United Republic of Tanzania, Ministry of Health, Community Development, Gender, Elderly and Children. Health Sector Strategic Plan V July 2021-June 2026. Available: https://mitu.or.tz/wp-content/uploads/2021/07/Tanzania-Health-Sector-Strategic-Plan-V-17-06-2021-Final-signed.pdf. Accessed: 27 July 2022.

[6] United Nations Human Settlements Programme (UN-Habitat). The State of African Cities: A framework for addressing urban challenges in Africa. Available: https://unhabitat.org/the-state-of-the-african-cities-report-2008. Accessed: 21 June 2022.

[7] Desgroppes A Taupin S Kibera: The Biggest Slum in Africa? East African Rev. 2011;44:23-33.

[8] Levira F Todd G Urban health in Tanzania: questioning the urban advantage. J Urban Health. 2017;94:437-49. 10.1007/s11524-017-0137-2 28303533 PMC5481212

[9] Mberu BU Haregu TN Kyobutungi C Ezeh AC Health and health-related indicators in slum, rural, and urban communities: a comparative analysis. Glob Health Action. 2016;9:33163. 10.3402/gha.v9.33163 27924741 PMC5141369

[10] Sanghera B Justice, power and informal settlements: Understanding the juridical view of property rights in Central Asia. Int Sociol. 2020;35:22-44. 10.1177/0268580919877596

[11] Rasmussen M. The power of Informal Settlements. The Case of Dar Es Salaam, Tanzania 2013. Available: http://www.hdm.lth.se/fileadmin/hdm/Education/Research/12_CTBT2012_by_Planum_no_26-2013_Rasmussen_Section_1-1__5_.pdf. Accessed: 21 July 2022.

[12] Nuhu S Munuo N Mngumi L Governance challenges of regularisation of informal settlements in peri-urban Tanzania: perspectives from local stakeholders. Int J Urban Sustain Dev. 2023;15:35-47. 10.1080/19463138.2023.2167821

[13] Kapologwe NA Meara JG Kengia JT Sonda Y Gwajima D Alidina S Development and upgrading of public primary healthcare facilities with essential surgical services infrastructure: a strategy towards achieving universal health coverage in Tanzania. BMC Health Serv Res. 2020;20:218. 10.1186/s12913-020-5057-2 32183797 PMC7076948

[14] Kapologwe NA Kalolo A Kibusi SM Chaula Z Nswilla A Teuscher T Understanding the implementation of Direct Health Facility Financing and its effect on health system performance in Tanzania: a non-controlled before and after mixed method study protocol. Health Res Policy Syst. 2019;17:11. 10.1186/s12961-018-0400-3 30700308 PMC6354343

[15] Yahya T Mohamed M Raising a mirror to quality of care in Tanzania: the five-star assessment. Lancet Glob Health. 2018;6:e1155-7. 10.1016/S2214-109X(18)30348-6 30196094

[16] Neiderud C-J How urbanisation affects the epidemiology of emerging infectious diseases. Infect Ecol Epidemiol. 2015;5:27060. 26112265 10.3402/iee.v5.27060 PMC4481042

